# Effects of Selected Physicochemical Parameters on Zerumbone Production of *Zingiber zerumbet* Smith Cell Suspension Culture

**DOI:** 10.1155/2015/757514

**Published:** 2015-02-12

**Authors:** Mahanom Jalil, Mohamad Suffian Mohamad Annuar, Boon Chin Tan, Norzulaani Khalid

**Affiliations:** ^1^Centre for Foundation Studies in Science, Faculty of Science, University of Malaya, 50603 Kuala Lumpur, Malaysia; ^2^Centre of Biotechnology for Agriculture Research (CEBAR), Faculty of Science, University of Malaya, 50603 Kuala Lumpur, Malaysia; ^3^Institute of Biological Sciences, Faculty of Science, University of Malaya, 50603 Kuala Lumpur, Malaysia

## Abstract

*Zingiber zerumbet* Smith is an important herb that contains bioactive phytomedicinal compound, zerumbone. To enhance cell growth and production of this useful compound, we investigated the growth conditions of cell suspension culture. Embryogenic callus generated from shoot bud was used to initiate cell suspension culture. The highest specific growth rate of cells was recorded when it was cultured in liquid Murashige and Skoog basal medium containing 3% sucrose with pH 5.7 and incubated under continuous shaking condition of 70 rpm for 16 h light and 8 h dark cycle at 24°C. Our results also revealed that the type of carbohydrate substrate, light regime, agitation speed, and incubation temperature could affect the production of zerumbone. Although the zerumbone produced in this study was not abundant compared to rhizome of *Z. zerumbet*, the possibility of producing zerumbone during early stage could serve as a model for subsequent improvement.

## 1. Introduction 

Plants represent an unlimited source of natural products, namely, primary and secondary metabolites. The needs of abundant and consistent qualities of planting materials are crucial to enable commercialization of the bioactive compounds. The current practice of harvesting bioactive compound needs a long period of plant cultivation and also facing problem with uncontrolled climatic conditions. Conventional farming also needs hectares of planting area to meet the purpose of providing a large amount of rhizomes for extraction. Due to that condition, it is important to have a model system to screen the bioactive compound production as early as in cell stage so that it could serve as an alternative source of material to harvest bioactive compounds continuously within a short period of time.

*Zingiber zerumbet* Smith is a perennial edible ginger with high phytomedical properties, commonly being used as herbal medicine in Asian, Indian, Chinese, and Arabic folklores since ancient time [1]. In* Z. zerumbet*, a cyclic sesquiterpene compound zerumbone (ZER) can be found abundantly in rhizomes (37%) followed by alpha-humulene and camphene [2]. ZER has a great demand due to its cosmeceutical properties for skin whitening and phytomedicinal properties such as antimicrobial and anti-inflammatory properties as well as curing chronic diseases such as cancer. ZER has been found to suppress tumor promoter cells [3] and also could inhibit the growth of human leukemia cell line, HL-60 cell [4]. Recently, it was also figured out that ZER could be used as chemopreventive agent in hepatocarcinogenesis [5]. Current study also pointed out that the ethanol extract of* Z. zerumbet* is not mutagenic and safe with respect to genotoxicity and general toxicity if individuals are provided with a proper dose [6].

Even though there are various works done on the extraction of ZER from* in vivo* and* in vitro* derived rhizome [7, 8], there was no report of ZER production from* in vitro* cell cultures. Realizing its huge potential in both medical and cosmetic industries and its crucial need of materials for extraction, this study was focused on the ZER production from cells cultures of* Z. zerumbet* Smith.

The aims of this study were to optimize the growth condition and to evaluate the production of ZER for* Z. zerumbet* Smith cell suspension cultures.

## 2. Materials and Methods

### 2.1. Initiation and Multiplication of Cell Suspensions

The callus of* Z. zerumbet* was initiated by culturing slices of shoot buds on Murashige and Skoog (MS) basal medium supplemented with 1.0 mg/L d-biotin, 2.0 mg/L 2,4-dichlorophenoxyacetic acid (2,4-D), 1 mg/L indoleacetic acid (IAA), and 1 mg/L 1-naphthaleneacetic acid (NAA) [8]. Cells suspension cultures were established by culturing friable callus in liquid MS medium containing 1 mg/L 2,4-D, 0.1 g/L malt extract, 0.1 g/L glutamine, 250 *μ*L/L zeatin, and 20 g/L sucrose. The media were adjusted to 5.7 prior to autoclaving. All cultures were maintained at 25 ± 1°C on orbital shaker at 70 rpm under a photoperiod of 16 h light and 8 h dark cycle with a light intensity of 31.4 *μ*mol m^−2^ s^−1^ provided by fluorescent lamps. The cell suspensions were subculture for every 7 days to multiply the cells.

### 2.2. Optimization of Growth Conditions for Cell Suspension Cultures

To optimize growth conditions for suspension cultures, carbohydrate substrates (2% sucrose, maltose, fructose, and glucose), different concentrations of sucrose (1%, 2%, and 3%), pH of medium (pH 5.2, 5.7, and 6.2), light regime (16 h light : 8 h dark and dark condition), agitation (40 rpm, 70 rpm, and 100 rpm), and incubation temperature (18, 24, and 30°C) were tested. The cells with initial volume of 0.5 mL settled cell volume (SCV) were inoculated into liquid MS media according to the experimental objectives. To obtain the SCV, cells were allowed to settle for 5 min and the fraction of the cell in liquid media was measured using graduated 15 mL Falcon tubes. The growth of cell suspension cultures was measured by SCV every 7 days for a period of 42 days whereas the specific growth rate (the changes of SCV in natural log) was recorded for 28 d of culture. All treatments are subjected to 2% of sucrose except for treatment with carbohydrate substrate which tested three other carbohydrate types. All media were adjusted to pH 5.7 prior to autoclaving and culture under continuous shaking condition at 70 rpm. All experiments were carried out in triplicate cultures.

### 2.3. Extraction of Zerumbone from Cells Suspension Culture

The cell suspension cultures were harvested after 15 days of initiation and oven-dried for 48 h at 38°C. Finely ground powder (0.1 g) was extracted using Soxhlet method for cells masses and 30 mL of filtrate of the liquid media undergoes partitioning. Crude extract was later evaporated by using rotary evaporator (BÜCHI Rotavapor R-114). The extract was dissolved in 10 mL dichloromethane (DCM, Merck, USA) and kept at 4°C in the chiller until use. Each analysis was repeated three times.

### 2.4. Identification of Zerumbone

The mixture was filtered through 0.45 *μ*m PTFE filter (Sartorius 13 CR), whereas liquid medium from cell suspension cultures was filtered using Whatman No. 1. An injection volume of 20 *μ*L was applied for each sample and elute was monitored at 254 nm in high performance liquid chromatography (HPLC) system (Waters, USA), consisting of a W600E multisolvent delivery system, W2489 UV/visible detector, W2707 autosampler and in-line degasser, guard, and reverse columns (Chromolith RP-18encapped, 100–4.6 mm), and W2707 autosampler controlled by Empower 2 software. The solvent for elution was 0.1% (v/v) phosphoric acid (A) and acetonitrile (B). The guard column and column were flushed with pure acetonitrile before and after use. The zerumbone compound was identified by matching its retention times (10.6–10.8 minutes) to commercially available standard zerumbone (Sigma, USA).

### 2.5. Statistical Analysis

All data collected were analyzed by one way ANOVA followed by Tukey's HSD test at a significance level of *P* < 0.01.

## 3. Results and Discussion

### 3.1. Establishment of Cells Suspension Culture

**Figure 1:**
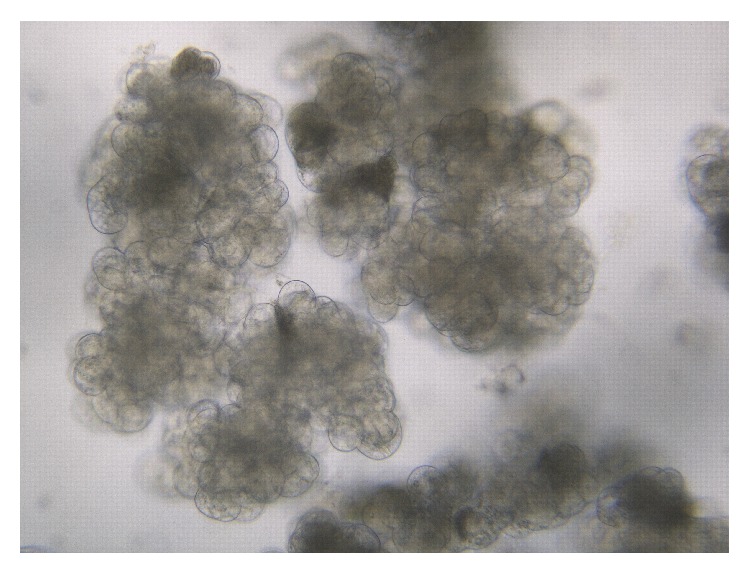
Homogenous cell suspension culture of* Zingiber zerumbet* Smith.

Friable callus obtained from the culture of slices of shoot buds was transferred into liquid medium for initiation of cell suspension culture. Heterogeneous cells containing dense cytoplasm were observed within a month of culture. The cells were filtered and continuously subcultured. Homogeneous cell suspension cultures with dense yellowish cytoplasm were obtained after 2-3 months of culture (Figure 1). Similar characteristics of viable cells were reported in banana cell suspension culture [9, 10].

### 3.2. Effect of Carbohydrate Substrate on Cells Biomass and ZER Production

**Figure 2:**
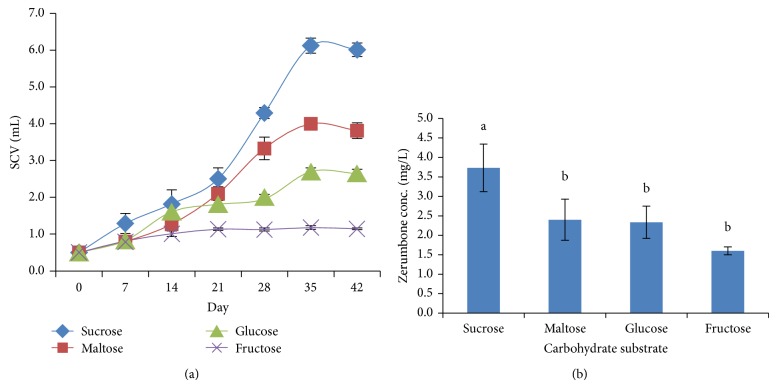
(a) Cells growth in different type of carbohydrate substrate. (b) Zerumbone production in different type of carbohydrate substrate.

Carbohydrate is one of the most important elements supplied into the media for the* in vitro* plant cells growth. In this study, we observed that the growth of cell suspension cultures was influenced by the carbohydrate substrate (Figure 2(a)). Of these, cells cultured in sucrose-based medium recorded the highest specific growth rate (0.0714) and zerumbone content (3.73 ± 0.61 mg/L), followed by maltose and glucose (Figure 2(b)). Low specific growth rate and zerumbone content were observed when cells were cultured in medium supplemented with fructose, indicating that fructose was less preferred for cell growth. In our study, production of ZER was also significantly affected by different carbohydrate substrate. Different carbon sources have been reported to influence the callogenesis and organogenesis [11]. Among available carbon sources, sucrose has been the major one [12]; however, it may cause hypoxia and ethanol accumulation in the cells due to its quick metabolization [13, 14]. Therefore, in some cases, sucrose is totally or partially replaced by other carbon source [15, 16].

### 3.3. Effect of Sucrose Concentrations on Cells Biomass and ZER Production

**Figure 3:**
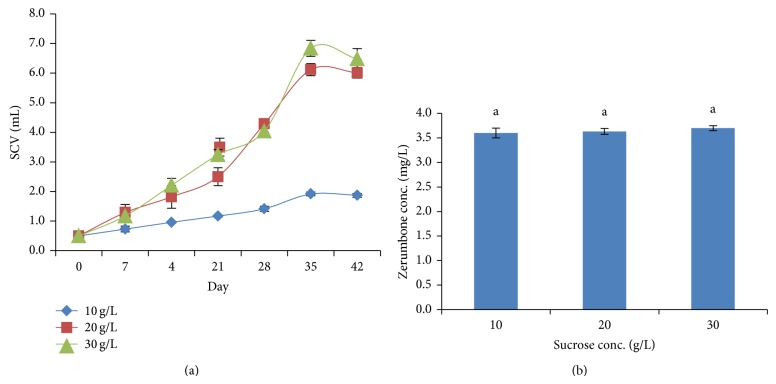
(a) Cells growth in different sucrose concentrations. (b) Zerumbone production in different sucrose concentrations.

In the subsequent experiment, we tested MS medium with different concentration of sucrose. Cells cultured in medium containing 2 and 3% sucrose showed almost similar specific growth rates (0.0715 and 0.0745, resp.) with doubling time at about 5-6 days of culture whereas medium with 1% sucrose showed the lowest growth rate with doubling SCV at 15–20 days of culture (Figure 3(a)). In contrast, the production of ZER was not significantly affected by the different concentrations of sucrose studied. The highest amount of ZER (3.70 ± 0.05 mg/L) was detected on medium containing 3% sucrose (Figure 3(b)). Sucrose served as a carbon source and regulator in cells osmosis [15]. Most* in vitro* cultures are autotrophic-incompetent and are not able to proliferate properly without exogenous supply of carbohydrates [17]. For the regeneration of* Digitalis lanata *Ehrh, the optimum concentration for all the carbohydrates tested (sucrose, maltose, fructose, and glucose) was 3.0 g/L [18]. Another study reported that the optimum concentration of sucrose for specific protease activity in culture medium was 262.8 mM [19]. While in* Rubia tinctorum* cultures, 350.4 mM sucrose should be used in order to produce maximum anthraquinone production [20]. However, high sucrose levels might cause cells dehydration and therefore reduced cells proliferation. This fact was supported by the finding that stated high sucrose concentration would reduce the shoot length in* Curcuma xanthorriza* and* Zingiber aromaticum* regeneration [21].

### 3.4. Effect of Initial pH Medium on Cells Biomass and ZER Production

**Figure 4:**
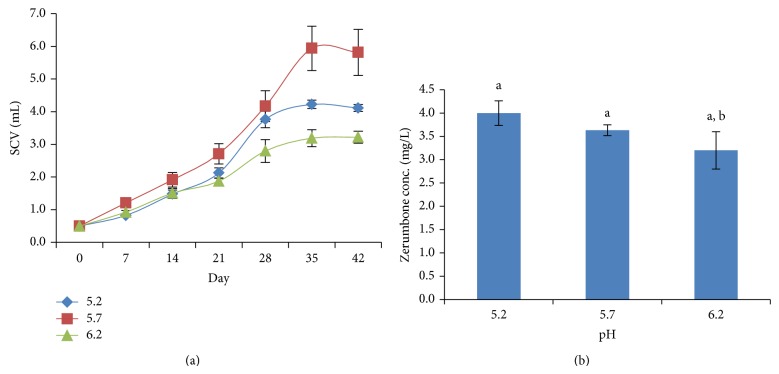
(a) Cells growth in different initial pH. (b) Zerumbone production in different initial pH.

Initial pH of medium prior to autoclaving was also investigated. Medium adjusted to pH5.7 recorded the highest specific growth rate of 0.0707 (Figure 4(a)). Increased medium pH from 5.7 to 6.2 was found to decrease the cells multiplication. As for zerumbone production, it was superior in media with initial pH 5.2 with the concentration of zerumbone at 4.00 ± 0.26 mg/L. However, medium with pH tested did not affect the production of ZER (Figure 4(b)). The medium pH is usually adjusted to between 5 and 6 before autoclaving and extremes of pH are avoided. It was also due to the need of preventing pH changes during the culture period by achieving an equilibration of medium pH before explants inoculation [22]. Another study revealed that lower (4.5–5) and higher (6.5–7.5) medium pH significantly reduced shoot height in tomato cv. Red Coat but callus production and growth remained unaffected. This suggested that the explants of tomato Cv. Red Coat are moderately tolerant to a wide range of medium pH, as most of the traits studied remained unaffected by the change in media pH [23]. However, the best regeneration and growth occurred only in the pH range 5.5–6.0. This fact was further supported by a study on the induction of friable callus from leaf explants of* Aquilaria malaccensis* for the purpose of establishing cell suspension culture which was highly effective at pH 5.7 [24].

### 3.5. Effect of Light Regime on Cells Biomass and ZER Production

**Figure 5:**
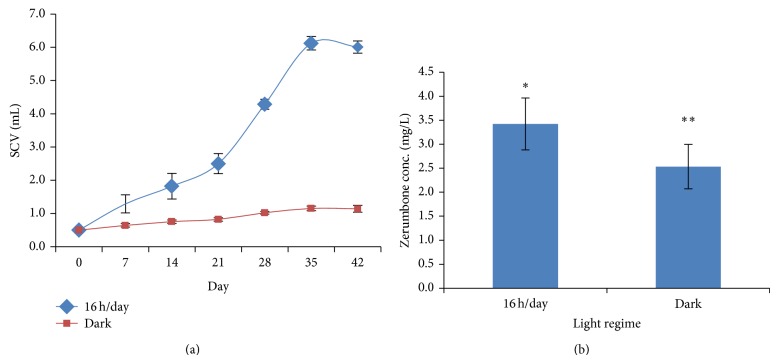
(a) Cells growth in different light regime. (b) Zerumbone production in different light regime.

Light regime is another factor affecting the cell growth and zerumbone production. Cells incubated under 16 : 8 (light : dark) condition were found to double the initial SCV on day 5 and produced the highest amount of ZER (3.42 ± 0.54 mg/L), whereas, under dark condition, SCV were only doubled on day 28 and produced 2.53 ± 0.46 mg/L ZER (Figures 5(a) and 5(b)). It was proved that irradiation had a remarkable effect on the growth of* Oldenlandia affinis* callus [25] while another scientist reported that the light quality played an important role in somatic embryogenesis in* Carica papaya* L. [26]. For the induction of rooting in* Eucalyptus saligna* Smith and* Eucalyptus globulus* Labill, it should be carried out in the dark for the first few days to increase the efficiency of the process [27]. In contrast,* in vitro* rooting of most conifers prefers a 16 h photoperiod [28].

### 3.6. Effect of Agitation on Cells Biomass and ZER Production

**Figure 6:**
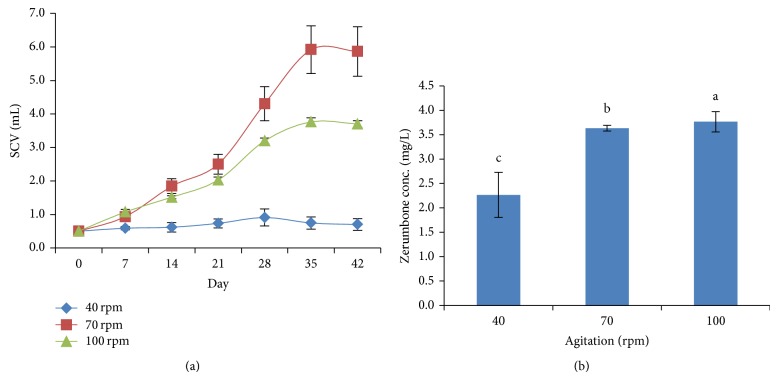
(a) Cells growth in different agitation. (b) Zerumbone production in different agitation.

Continuous agitation is another important parameter for growing cell suspension cultures. Agitation at 70 and 100 rpm was found to support the growth of* Z. zerumbet* cells suspension culture. Minimum cell growth was observed when agitated at 40 rpm compared to others (Figure 6(a)). In terms of zerumbone production, cells grown under continuous shaking condition of 70 and 100 rpm were able to produce more than 3.60 mg/L ZER (Figure 6(b)). This might be due to the increase in cells stress condition due to higher speed of agitation. The study on palm oil suspension culture showed more than 200% increment of biomass obtained from initial inoculum at high agitation rates (120 and 225 rpm) in bioreactor system [29].

### 3.7. Effect of Temperature on Cells Biomass and ZER Production

**Figure 7:**
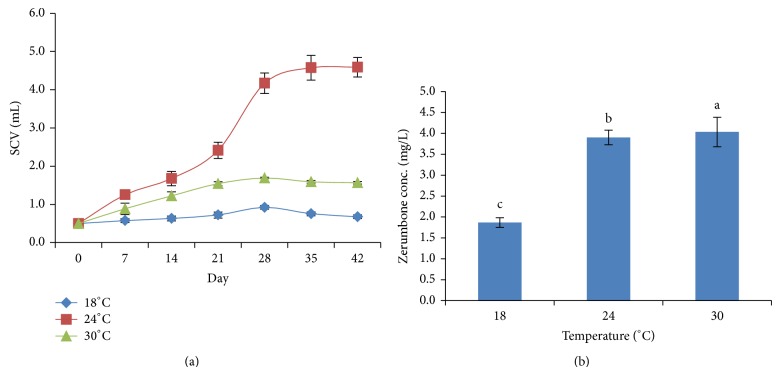
(a) Cells growth in different incubation temperature. (b) Zerumbone production in different incubation temperature.

In this study, we also investigated the effect of temperature on cell growth and ZER production. Our results revealed that low temperature (18°C) negatively affected the cell growth and ZER production (Figures 7(a) and 7(b)). At 24°C, cell growth was drastically increased after 21 days of culture with production of 3.90 mg/L ZER. Only 1.87 mg/L ZER was detected in cells incubated at 18°C. Temperature ranging from 17 to 25°C is normally used for induction of callus tissues and growth of plant cells culture but each plant species may favour a different temperature. Previous study stated that lowering the cultivation temperature increased the total fatty acid content per cell in dry weight in* Catharanthus roseus* cells suspension culture [30]. Further investigation concluded that the optimum temperature for plants cell growth was at 24°C while paclitaxel synthesis was produced at maximum rate at 29°C [31].

## 4. Conclusion

In summary, medium with different composition was found to affect the growth of* Z. zerumbet* cell suspension culture as well as the production of ZER. Production of secondary metabolite might be influenced by the viability of cells and culture conditions. It is worth noting that ZER was found secreted into spent liquid medium and only some residue of ZER detected in the cells biomass. Although zerumbone produced was not as abundant compared to rhizome, but the possibility of producing zerumbone during early stage could serve as a model to improve secondary compound metabolites using cell culture.
